# Cross-Regulation between Oncogenic BRAF^V600E^ Kinase and the MST1 Pathway in Papillary Thyroid Carcinoma

**DOI:** 10.1371/journal.pone.0016180

**Published:** 2011-01-13

**Authors:** Seong Jin Lee, Min Hee Lee, Dong Wook Kim, SeongEun Lee, Songmei Huang, Min Jeong Ryu, Yong Kyung Kim, Sung Jin Kim, Soung Jung Kim, Jung Hwan Hwang, Sangphil Oh, Heeyeong Cho, Jin Man Kim, Dae-Sik Lim, Young Suk Jo, Minho Shong

**Affiliations:** 1 Department of Internal Medicine, Chungnam National University School of Medicine, Daejeon, Republic of Korea; 2 Department of Pathology, Chungnam National University School of Medicine, Daejeon, Republic of Korea; 3 Pharmacology Research Center, Korea Research Institute of Chemical Technology, Daejeon, Republic of Korea; 4 Animal Model Center, Korea Research Institute of Bioscience and Biotechnology, Daejeon, Republic of Korea; 5 Department of Biological Sciences, Korea Advanced Institute of Science and Technology, Daejeon, Republic of Korea; 6 Research Center for Endocrine and Metabolic Diseases, Chungnam National University Hospital, Daejeon, Republic of Korea; The National Institute of Diabetes and Digestive and Kidney Diseases, United States of America

## Abstract

**Background:**

The BRAF^V600E^ mutation leading to constitutive signaling of MEK-ERK pathways causes papillary thyroid cancer (PTC). Ras association domain family 1A (RASSF1A), which is an important regulator of MST1 tumor suppressor pathways, is inactivated by hypermethylation of its promoter region in 20 to 32% of PTC. However, in PTC without RASSF1A methylation, the regulatory mechanisms of RASSF1A-MST1 pathways remain to be elucidated, and the functional cooperation or cross regulation between BRAF^V600E^ and MST1,which activates Foxo3,has not been investigated.

**Methodology/Principal Findings:**

The negative regulators of the cell cycle, p21 and p27, are strongly induced by transcriptional activation of FoxO3 in BRAF^V600E^ positive thyroid cancer cells. The FoxO3 transactivation is augmented by RASSF1A and the MST1 signaling pathway. Interestingly, introduction of BRAF^V600E^markedly abolished FoxO3 transactivation and resulted in the suppression of p21 and p27 expression. The suppression of FoxO3 transactivation by BRAF^V600E^is strongly increased by coexpression of MST1 but it is not observed in the cells in which MST1, but not MST2,is silenced. Mechanistically, BRAF^V600E^was able to bind to the C-terminal region of MST1 and resulted in the suppression of MST1 kinase activities. The induction of the G1-checkpoint CDK inhibitors, p21 and p27,by the RASSF1A-MST1-FoxO3 pathway facilitates cellular apoptosis, whereasaddition of BRAF^V600E^ inhibits the apoptotic processes through the inactivation of MST1. Transgenic induction of BRAF^V600E^in the thyroid gland results in cancers resembling human papillary thyroid cancers. The development of BRAF^V600E^transgenic mice with the MST1 knockout background showed that these mice had abundant foci of poorly differentiated carcinomas and large areas without follicular architecture or colloid formation.

**Conclusions/Significance:**

The results of this study revealed that the oncogenic effect of BRAF^V600E^ is associated with the inhibition of MST1 tumor suppressor pathways, and that the activity of RASSF1A-MST1-FoxO3 pathways determines the phenotypes of BRAF^V600E^ tumors.

## Introduction

Activating mutations in the BRAF gene are found at high frequency in various human cancers, and BRAF^V600E^ is the most common of these activating mutations, especially in papillary thyroid cancer, where it is found at a frequency of 40–70% [1], [2], [3]. In BRAF^V600E^-positive thyroid cancer cell lines and BRAF^V600E^ transgenic mice, this mutation is responsible for tumor initiation, transformation, growth, proliferation and dedifferentiation [4], [5], [6]. Research into the molecular mechanisms of BRAF^V600E^-positive tumors has revealed that the missense valine to glutamic acid mutation increases kinase activity, promoting the constitutive activation of MEK-ERK signaling [7], [8], [9], [10], [11] and enhancing ERK-dependent transcriptional output [12], [13]. However, other signaling pathways except MEK-ERK [14], [15]regulated in BRAF^V600E^ tumors are not fully characterized [16]. Moreover, tumor suppressor systems which may be controlled by BRAF^V600E^ in thyroid cancer remain to be identified.

The tumor suppressor gene RASSF1A (Ras association domain family 1A) is epigenetically inactivated through *de novo* promoter methylation in the early stages of thyroid tumorigenesis [17], [18]. Interestingly, RASSF1A has recently been described as an important activator of MST1, which in turn phosphorylates and promotes the nuclear translocation of the forkhead transcription factor FKHRL1 (FoxO3), inducing cell death [19], [20], [21]. This suggests that FoxO3 transactivation could be induced by the RASSF1A-MST1 pathway and function as a tumor suppressor system in response to specific oncogenic signals, such as BRAF^V600E^. However, promoter hypermethylation of RASSF1A could only be detected in a relatively small percentage of PTC (20 to 32%) [18], [22]. These observations predict that novel RASSF1A-MST1-FoxO3 pathways regulated by BRAF^V600E^ might function during the development of PTC whichdoes nothave RASSF1Apromoter methylation.

FoxO3 transactivation is effectively inhibited by RET/PTC (rearranged in transformation/papillary thyroid carcinomas) kinase [23], the gene rearrangement of which is the most common rearrangement in papillary thyroid cancer. The inactivation of FoxO3 could therefore be a signature molecular event that should also occur in BRAF^V600E^ thyroid tumors. Several molecular mechanisms which are possibly regulated by BRAF^V600E^ may control FoxO3 activity in thyroid cancer. First, as RET/PTC kinase inhibits FoxO3 transactivation through an Akt/PKB dependent pathway, BRAF^V600E^ might also activate Akt/PKB signaling pathway [24]. Second, the constitutive activation of ERK by BRAF^V600E^ could inhibit FoxO3 activation via the ubiquitin-proteasome pathway [25]. Finally, BRAF^V600E^ could act through crosstalk with the RASSF1A-MST1-FoxO3 pathway.

Based on these hypotheses, we decided to investigate the regulation of the MST1-FoxO3 pathway by BRAF^V600E^. This resulted in the identification of novel cross-talksignaling between BRAF^V600E^ and MST1, thereby demonstrating the functional activity of the RASSF1A-MST1-FoxO3 tumor suppressor system. Furthermore, *in vivo* experiments showed that MST1 knockout mice exhibited more aggressive BRAF^V600E^ tumor phenotypes.

## Materials and Methods

### Plasmids

The pCMV5-Myc-FoxO3 plasmid was purchased from Addgene (Addgene Inc., Cambridge, MA) and pcDNA3.1/CT-GFP-FoxO3 was constructed using the CT-GFP Fusion TOPO Expression Kit according to the manufacturer's protocol (Invitrogen, Carlsbad, CA). Expression vectors (pME18) for Flag epitope-tagged forms of human MST1, MST1-N (residues 1–326), MST1-C (residues 327–487), HA-RASSF1A and 3X-IRS reporters have been described previously [26], [27], [28], [29]. The cDNA for human BRAF was cloned into pLenti6/V5-DEST using the pDONR221 vector (Invitrogen). For the cloning of BRAF, PCR primers were designed as follows: sense, 5′-GGGGACAAGTTTGTACAAAAAAGCAGGCTTCAAGATGGCGGCGCTGAGCGGT-3′, and antisense, 5′- GGGGACCACTTTGTACAAGAAAGCTGGGTCTCAGTGGACAGGAAACGCCC-3′. To generate vectors for human BRAF^V600E^ and BRAF^G469A^, the Site-Directed PCR Mutagenesis Kit (Stratagene, La Jolla, CA) was used to introduce the missense mutation into the wild-type BRAF sequence. SiRNAs for MST1 and RAF-1 were purchased from Invitrogen (Stealth RNAi, Invitrogen).

### Cell culture and transfection

FRO cells and 293T cells were cultured in RPMI and DMEM supplemented with 10% fetal bovine serum [20], [30]. Cells (2×10^5^/35 mm dish) were transfected with siRNA at a concentration of 50 nM using the HiPerfect Transfection Reagent (Qiagen, Valencia, CA) and/or with the indicated vectors using Fugen6 Transfection Reagent (Roche Applied Science, Indianapolis, IN). The MEK inhibitor (U0126) and PI3K inhibitors (Ly294002, Wortmannin) were purchased from Cell Signaling Technology (Beverley, MA).

### Antibodies

Primary antibodies against p21, p27, phospho-ERK, total ERK, phospho-Akt, total Akt, MST1, Actin, FoxO3, Myc (Cell Signaling), BRAF (C-19, Santa Cruz Biotechnology, Santa Cruz, CA), RASSF1A (eBioscience, San Diego, CA) and Flag (Sigma, St Louis, MO) were purchased from the indicated companies.

### Luciferase-based reporter assay

To assay the activity of FoxO3, 293T cells were transfected with 3X-IRS-Luc, or p27-Luc and pRL-SV40, which encodes *Renilla* luciferase (Promega, Madison, WI) using Lipofectamine in OptiMEM (Invitrogen). Luciferase levels were determined using the Dual Luciferase Assay Kit (Promega). Firefly and *Renilla* luciferase activities were measured sequentially from the same sample using a Fusion Alpha-Microplate Analyzer (Perkin Elmer, Waltham, MA). For each sample, firefly luciferase activity was normalized to *Renilla* luciferase activity.

### Immunoprecipitation and Immunoblot analysis

All immunoprecipitation and immunoblotting procedures were performed at 4°C. Cells were washed twice with PBS and lysed in lysis buffer containing protease inhibitors. Lysates were centrifuged at 14,000 g for 15 min at 4°C. For the IP analysis, lysates were precleared with protein A/G beads (Santa Cruz Biotechnology) for 30 min, and supernatants were incubated with the indicated primary antibody for 3 h with agitation. Protein A/G beads were then added to the mixture and incubated for at least 2 h at 4°C. Beads were washed with lysis buffer and the immunoprecipitates were subjected to immunoblotting. Cell lysates were separated by SDS-PAGE, and transferred to nitrocellulose membranes. Membranes were blocked with TBS containing 5% milk and 0.1% Tween20 for 1 h and then incubated overnight with primary antibody at 4°C. Membranes were washed in TBS/T and incubated with secondary antibodies conjugated to horseradish peroxidase (Cell Signaling) for 1 h at RT. Blots were developed using the LumiGLOChemiluminescent substrate (Cell Signaling).

### Immunofluorescence staining

293T cells were grown on 6-well plates and transfected with the indicated plasmids using LipofectAmine (Invitrogen). Twenty-four hours after transfection, the cells were washed three times with PBS and fixed in 3.7% formaldehyde for 20 min. Cells were permeabilized with PBS containing 0.1% Triton X-100 and 0.1 M glycine at RT, incubated for 15 min, washed three times with PBS, and blocked with 3% BSA in PBS for 10 min at RT. Cells were incubated with primary anti-Myc and anti-Flag for 1 h at 37°C, washed three times with PBS, and incubated for 1 h at 37°C with Cy5-conjugated anti-Rabbit and TRITC-conjugated anti-Mouse for Myc-BRAF^V600E^ and Flag-MST1, respectively (Jackson ImmunoResearch Laboratories, West Grove, PA). Cells were observed with a CellomicsArrayScanV HCS reader (Thermo Scientific, Pittsburgh, PA).

### *In vitro* kinase assay of MST1

MST1 was immunoprecipitated from cell lysates and the washed precipitates were incubated for 30 minutes at 30°C with 1 µg histone H2B (Roche) in 25 µL kinase assay buffer comprised of 40 mM HEPES-NaOH (pH 7.4), 20 mM MgCl_2_, 1 mM DTT, phosphatase inhibitor mixture, 10 µM unlabeled ATP, and 1 µCi [γ-32P]ATP. The reaction was terminated by the addition of Laemmli sample buffer and phosphorylated proteins were detected by SDS-PAGE followed by autoradiography.

### Apoptosis Assay

Image based apoptosis assays were carried out using the APO-BrdU TUNEL Assay Kit (Invitrogen). Cells was transfected with FoxO3, MST1, and/or BRAFV^600E^, as indicated, and then stained with DAPI and terminal deoxynucleotidyltransferase–mediated dUTP nick end labeling (TUNEL) reagents.The proportion of apoptotic cells was determined asthe percentage of TUNEL-positive cells among all DAPI-stainedcells.

### Mouse Experiments and Histology

The strategy and methods used for the generation of thyroid specific transgenic BRAF^V600E^ FVB/N mice with a bovine thyroglobulin promoter have been described previously [4]. A detailed explanation of how MST1 null mice are generated is described in a recent paper from our group [31]. The MST1-null mice appeared to have normal reproductive capacity when compared with wild-type mice. Tg-BRAF^V600E^ mice were crossed with MST1-null mice to obtain Tg-BRAF^V600E^/MST1-null mice and Tg-BRAF^V600E^ littermates. These mice were sacrificed at 16 weeks and their thyroids were removed after genotyping. Thyroid tissues were fixed in 10% formalin and embedded in paraffin. Five µM-thick sections were prepared and stained with H&E. Immunohistochemical analysis of paraffin embedded thyroid tissue was also performed using anti-cyclin D1 rabbit monoclonal antibody (92G2, Cell Signaling). ImageJ (http://rsbweb.nih.gov/ij/) for microscopy was used to estimate areas without follicular architecture or colloid formation in thyroid tissues. Tg-BRAF^V600E^/MST1 null mice (n = 10) and Tg-BRAF^V600E^ littermates (n = 10) were collected and three sections of each mouse were used for immunohistochemical and imageJ analysis. Differences in the number of undifferentiated foci and areas without follicular architecture or colloid formation between both groups were assessed by the Mann-Whitney U-test. All animal experiments were approved by the Institutional Animal Care and Use Committee of the Chungnam National University School of Medicine (Approval ID; 2009-3-25).

### Statistical Analysis

Results were expressed as the means ± standard deviations. Statistical differences between two groups were analyzed by the Mann-Whitney U test. Differences were considered to be significant at *P*<0.05.All statistical analyses were performed using SPSS Version 16.0 for Windows (SPSS Inc., Chicago, IL).

## Results

### BRAF^V600E^ suppresses p21 and p27 induction by RASSF1A in FRO cells

**Figure 1:**
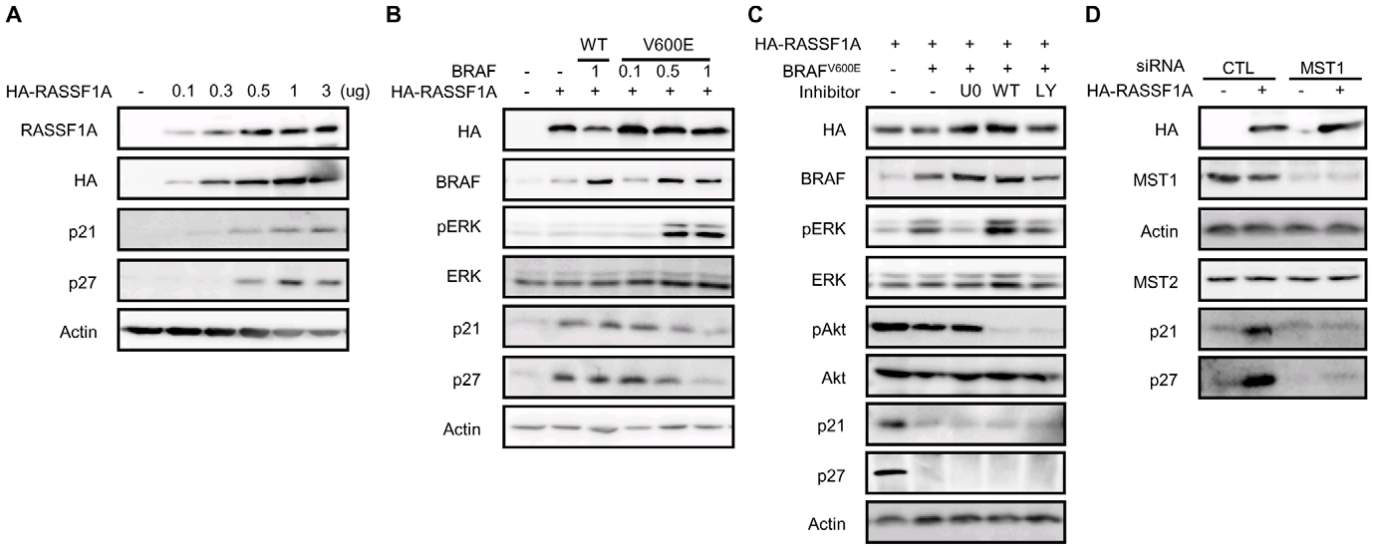
BRAF^V600E^ suppresses p21 and p27 induction by RASSF1A in FRO cells. (a) FRO Cells were transfected with HA-RASSF1A as indicated and incubated for 24 h. Total lysates were analyzed for p21 and p27 expression by Western blot analysis. (b) Lysates from FRO cells co-transfected with vectors for HA-RASSF1A and BRAF (wild type or the V600E mutant), as indicated, were subjected to Western blot analysis. (c) FRO cells transiently transfected with BRAF^V600E^ and RASSF1A were incubated with MEK (U0126, 10 µM) or PI3K (wortmannin, 1 µM, Ly294002, 10 µM) inhibitors for 12 h as indicated. The lysates were subjected to western blot analysis with primary antibodies as indicated. (d) FRO cells co-transfected with RASSF1A (0.5 µg/well) and SiMST1 (20 µM/well Stealth™ RNA) were incubated for 24 hrs as indicated. Abbreviations: CTL; control.

To confirm that RASSF1A induces FoxO3 transactivation, RASSF1A was transiently transfected into FRO cells harboring the BRAF mutated alleles and then p21 and p27, the known transcriptional targets of FoxO3, were assessed by immunoblotting [30]. As shown in Fig 1A, endogenous RASSF1A was not detected, which is compatible with previous reports showing hypermethylation of RASSF1A in undifferentiated thyroid cancer cell lines [17], [32]. Fig. 1A shows the RASSF1A-induced dose dependent increase in p21 and p27, which suggests that RASSF1A might be linked with FoxO3 transactivation. To investigate the possible crosstalk between RASSF1A and BRAF^V600E^, the effect of BRAF^V600E^ on RASSF1A-mediated p21 and p27 induction was evaluated. Interestingly, BRAF^WT^ did not affect the induction of p21 and p27 by RASSF1A, whereas BRAF^V600E^ effectively decreased p21 and p27 (Fig. 1B), suggesting that oncogenic BRAF^V600E^ can regulate the RASSF1A-FoxO3 signaling pathway. As BRAF^V600E^ can inhibit FoxO3 transactivation via the ERK or Akt/PKB signaling pathways, a MEK inhibitor (UO126) and PI3K inhibitors (Wortmannin, LY294002) were used to block these pathways and observe the effect of BRAF^V600E^ on RASSF1A-induced FoxO3 transactivation. As shown in Fig. 1C, the BRAF^V600E^-mediated suppression of p21 and p27 induction by RASSF1A was not reversed by the MEK inhibitor U0126 or the PI3K inhibitors Wortmannin and LY294002, showing that BRAF^V600E^ inhibits the RASSF1A-induced p21 and p27 upregulation independently from the ERK and PI3K-Akt/PKB signaling pathways. Recently, RASSF1A was shown to interact with MST1 via its SARAH domain and increase MST1 kinase activity [20], suggesting that RASSF1A-MST1-FoxO3 might be operational in response to oncogenic signals. To verify this hypothesis, MST1 expression was silenced using siRNA, and the RASSF1A induction of p21 and p27 was assessed. Fig. 1D shows that RASSF1A did not induce p21 and p27 expression in siMST1-treated cells, indicating that MST1 plays an important role in the p21 and p27 induction by RASSF1A.

### BRAF^V600E^ represses FoxO3 transactivation by the RASSF1A-MST1 signaling pathway

**Figure 2:**
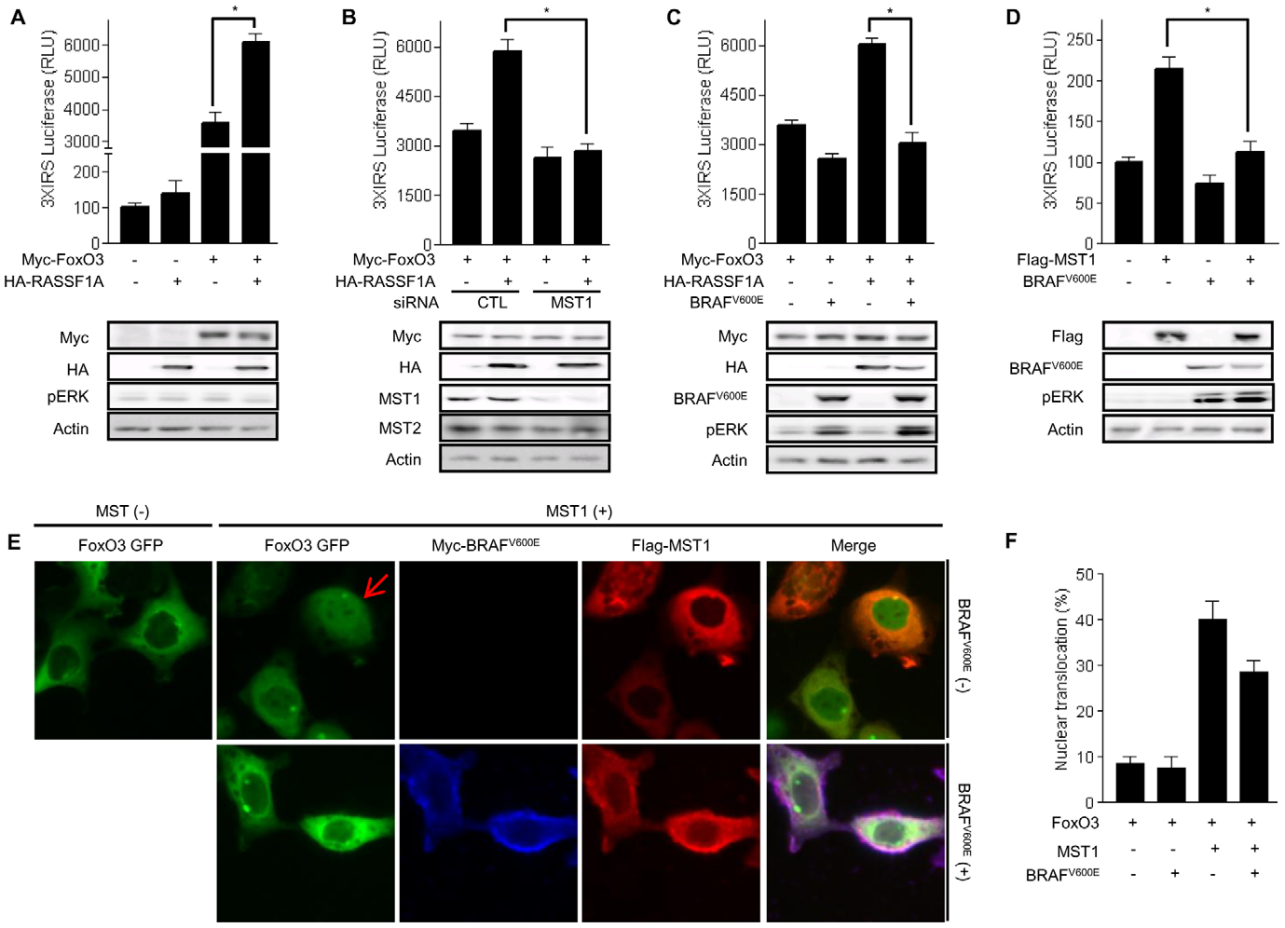
BRAF^V600E^ represses FoxO3 transactivation induced by RASSF1A-MST1 signal. 293T cells were transfected as indicated and luciferase-based reporter assays were conducted to observe 3XIRS reporter activity. For each sample, firefly luciferase activity was normalized to *Renilla* luciferase activity and expressed as the relative-fold change compared to basal luciferase activity. (a) Co-transfection with 3X-IRS Luc (100 ng/well), FoxO3 (0.5 µg/well), and RASSF1A (0.5 µg/well) was performed as indicated, and incubation lasted for 24 h. Total lysates were immunoblotted with anti-Myc, anti-HA, or anti-Actin antibodies. (b) Co-transfection with 3XIRS Luc (100 ng/well), FoxO3 (0.5 µg/well), RASSF1A (0.5 µg/well), and SiMST1 (20 µM/well Stealth™ RNA) was performed as indicated, and incubation lasted for 24 h. Total lysates were immunoblotted with anti-Myc, anti-HA, anti-MST, or anti-Actin antibodies. (c) Co-transfection with 3X-IRS Luc (100 ng/well), FoxO3 (0.5 µg/well), RASSF1A (0.5 µg/well), and BRAF^V600E^ (0.5 µg/well) was performed as indicated, and incubation lasted for 24 hours. Total lysates were immunoblotted with anti-Myc, anti-HA, anti-BRAF, or anti-Actin antibodies. (d) Co-transfection with 3X-IRS Luc (100 ng/well), MST1(0.5 µg/well), and BRAF^V600E^ (0.5 µg/well) was performed as indicated, and incubation lasted for 24 hours. All data are presented as mean±SD. Total lysates were immunoblotted with anti-Flag, anti-BRAF, or anti-Actin antibodies. (e) BRAF^V600E^ inhibits MST1-induced nuclear translocation of FoxO1. 293T cells were cultured in a six-well dish until they reached 80% confluence and co-transfected with FoxO3-GFP (0.5 µg/well), Flag-MST1 (0.5 µg/well), and Myc-BRAF^V600E^ (0.5 µg/well) as indicated. Twenty-four hours after transfection, the cells were fixed and incubated with antibodies against Myc or Flag, washed, and incubated with Cy5-conjugated anti-Rabbit for Myc-BRAF^V600E^ and TRITC-conjugated anti-Mouse for Flag-MST1. (f) Fluorescence was observed using a Cellomics ArrayScanV HCS reader. To calculate FoxO3 distribution, at least one thousand cells in each experiment were counted and analyzed by Cellomics BioApplication. All data are presented as mean±SD: (*) P<0.01 between two groups, Abbreviations: CTL; control.

The transcriptional activity of FoxO3 is regulated by posttranslational modifications such as phosphorylation, acetylation, nitrosylation and sumoylation [33], [34], [35].Among these modifications, the MST1-mediated serine 207 phosphorylation of FoxO3 is critical for its nuclear translocation [19], [36]. To investigate whether the RASSF1A-MST1 signal increased FoxO3 transactivation, a 3XIRS reporter system was used, consisting of a 3XIRS promoter construct containing three insulin response sequences/FoxO response elements (CAAAA[C/T]AA, the 3XIRS promoter) [26], [29]. Transient expression of MST1 significantly increased 3XIRS reporter activities in a dose-dependent manner (Fig. S1). Evaluation of the effect of RASSF1A on FoxO3 reporter activity showed that RASSF1A remarkably increased 3XIRS reporter activity (Fig. 2A). Moreover, this RASSF1A-induced FoxO3 reporter activation was completely abolished by MST1 silencing, indicating that RASSF1A effectively increased FoxO3 transactivation through a MST1-dependent pathway (Fig. 2B).

Because BRAF^V600E^ inhibited RASSF1A-induced p21 and p27 upregulation, the effect of BRAF^V600E^ on FoxO3 transactivation by RAASF1A was assessed. As shown in Fig. 2C, BRAF^V600E^ abrogated the RASSF1A-induced FoxO3 transactivation. Furthermore, MST1-induced FoxO3 transactivation was also repressed by BRAF^V600E^, indicating that BRAF^V600E^ inhibits the RASSF1A-MST1-FoxO3 pathway (Fig. 2D).

Because the transcriptional activity of FoxO3 depends on its nuclear translocation, a FoxO3-GFP vector was constructed to confirm the data obtained with the 3XIRS reporter by immunofluorescence imaging. As shown in Fig. 2E, GFP-fluorescence was predominantly observed in the cytoplasm in control samples; introduction of MST1 caused a shift in GFP-fluorescence detection to the nucleus. When BRAF^V600E^ was co-transfected with MST1, nuclear GFP fluorescence was barely detected. Interestingly, BRAF^V600E^ was exclusively localized in the cytoplasm (blue), MST1 showed dominant cytoplasmic localization (red), and merged images revealed a combined color (lilac) that suggests the co-localization of BRAF^V600E^ and MST1. To quantify FoxO3-fluorescence, the nuclear GFP-fluorescence signal intensity was analyzed using the BioApplication of CellomicsArrayScan, following the manufacturer's protocol. In agreement with the immunofluorescence images, MST1 increased intensity of the nuclear GFP-fluorescence signal, and BRAF^V600E^ inhibited the nuclear detection of GFP-fluorescence (Fig. 2F). Furthermore, we performed fractionation assay to define the subcellular localization of FoxO3-GFP and observed that MST1 increased nuclear FoxO3-GFP and BRAF^V600E^ could reverse this MST1-induced FoxO3 nuclear localization (Fig. S2).Taken together, these results indicate that BRAF^V600E^ inhibits nuclear translocation of FoxO3 and suggest that this inhibition might be mediated by direct interaction between BRAF^V600E^ and MST1.

### BRAF^V600E^ represses FoxO3 transactivation via MST1 kinase-dependent pathway

RAF-1 is an homologue of BRAF that interacts with MST2, suppressing MST2-mediated apoptosis [37], [38], which may be important for the regulation of certain biological processes [39]. In our study, however, FoxO3 inhibition by BRAF^V600E^ was not altered by RAF-1 silencing (Fig. S3). Furthermore, this inhibitory effect of BRAF^V600E^ was not reversed by the inhibition of MEK or PI3-kinase (Fig. S4), confirming that BRAF^V600E^ is able to suppress FoxO3 transactivation through a mechanism independent from MEK/ERK and PI3-kinase signaling.

**Figure 3:**
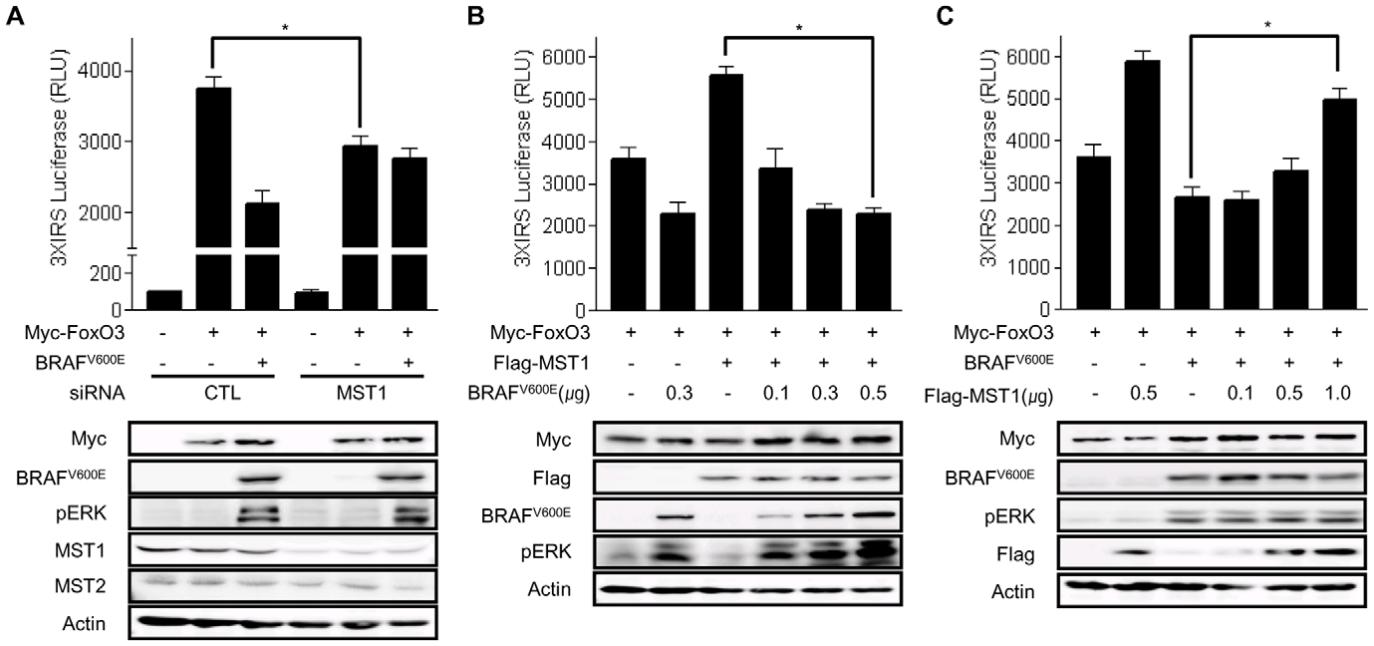
BRAF^V600E^ and MST1 kinases competitively regulate FoxO3 transactivation. (a∼c) Co-transfection with 3X-IRS Luc (100 ng/well), FoxO3 (0.5 µg/well), BRAF^V600E^ (indicated amount/well), MST1 (indicated amount/well), and/or SiMST1 (20 µM/well Stealth™ RNA) was performed as indicated. Twenty-four hours after transfection, a luciferase-based reporter assay was conducted. Total lysates were immunoblotted with anti-Myc, anti-HA, anti-MST1, anti-BRAF, anti-Flag, or anti-Actin antibodies. All data are presented as mean±SD: (*) P<0.01 between two groups, Abbreviations: CTL; control.

The inhibition of RASSF1A or MST1-induced FoxO3 transactivation and nuclear translocation by BRAF^V600E^led to the hypothesis that BRAF^V600E^ might regulate FoxO3 transactivation via a MST1-dependent pathway. To verify this hypothesis, reporter experiments were carried out in siMST1-treated cells. Remarkably, following knockdown of MST1, FoxO3 could not induce full activation of the 3XIRS reporter and BRAF^V600E^ no longer had an effect on FoxO3 induced activation of 3XIRS reporter (Fig. 3A). These observations suggest that MST1 plays a role in FoxO3 transactivation and that BRAF^V600E^ inhibits FoxO3 transactivation by suppressing MST1 activity. The reporter assay was also used to test whether there is signal-crosstalk between MST1 and BRAF^V600E^ on FoxO3 transactivation. As expected, BRAF^V600E^ inhibited MST1-induced 3XIRS reporter activation in a dose-dependent manner and MST1 abolished the inhibitory effect of BRAF^V600E^ on FoxO3 transactivation (Fig. 3B & 3C). These reporter assays indicated that increased levels of MST1 could prevent BRAF^V600E^-induced FoxO3 inactivation. Taken together, these data suggest that the cross-regulation between BRAF^V600E^ and MST1 determines the degree of FoxO3 transactivation.

### BRAF^V600E^ shows mutation-specific binding with the C-terminal of MST1 and inhibits MST1 kinase activity *in vitro*

**Figure 4:**
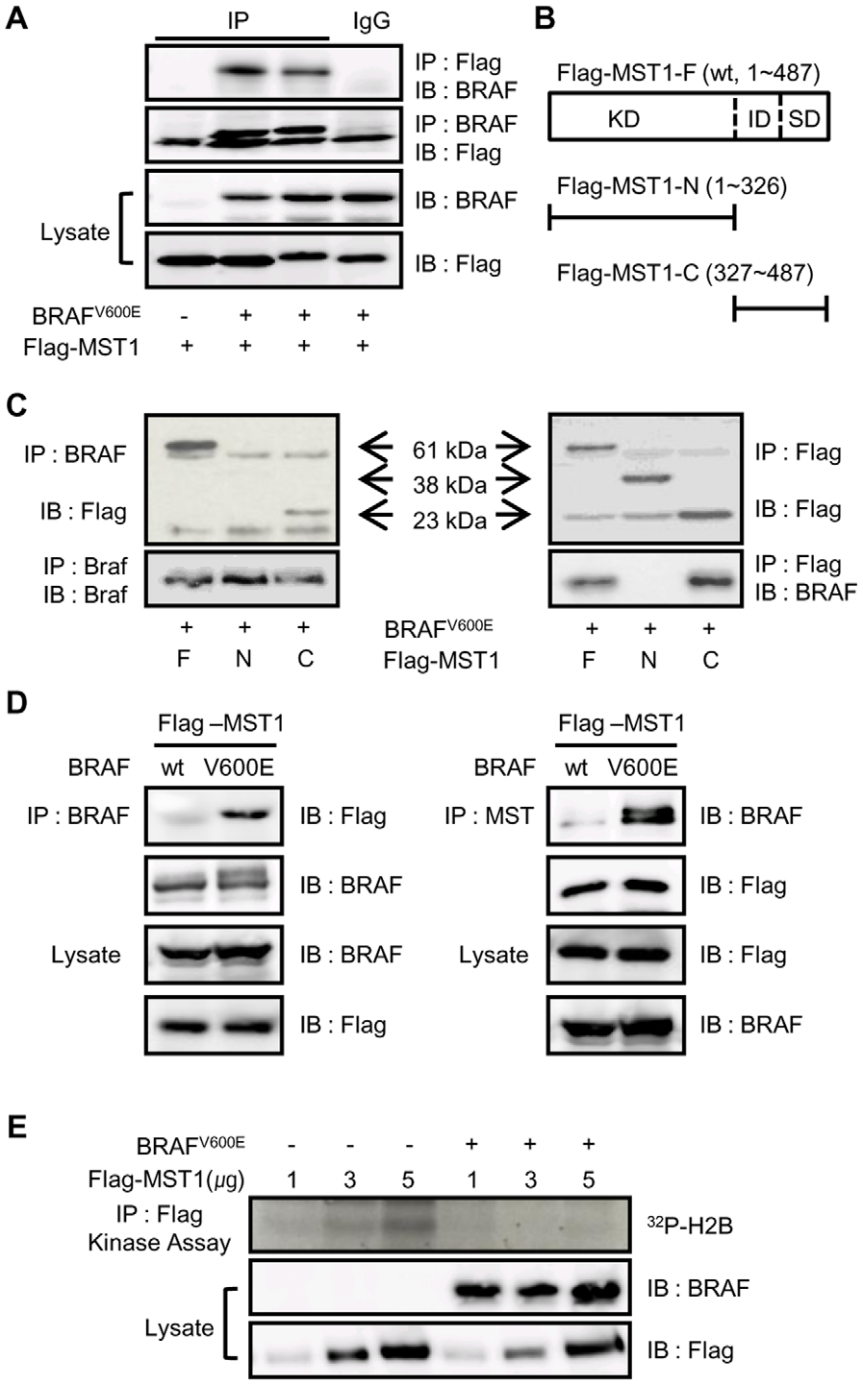
BRAF^V600E^ directly binds to the C-terminal domain of MST1 and represses MST1 kinase activity *in vitro*. (a–d) Co-transfection with BRAF^V600E^ (1 µg/well), BRAF^WT^ (1 µg/well), Flag-MST1 (1 µg/well), Flag-MST1-N (1 µg/well), and Flag-MST1-C (1 µg/well) was performed as indicated. Twenty-four hours after transfection, cells were lysed and the lysates were used for immunoprecipitation analyses with the indicated primary antibodies. The immunoprecipitates were separated by SDS-PAGE and subjected to immunoblotting with anti-Flag or anti-BRAF antibodies. (e) Co-transfection with BRAF^V600E^ (1 µg/well) and Flag-MST1 (indicated amount/well) was performed. Twenty-four hours after transfection, immunoprecipitation of MST1 from cell lysates was performed and the washed precipitates were incubated for 30 minutes at 30°C with 1 µg histone H2B in 25 µL kinase assay buffer. The reaction was terminated by the addition of Laemmli sample buffer and phosphorylated proteins were detected by SDS-PAGE followed by autoradiography.

Because immunofluorescence images and reporter assays suggested that BRAF^V600E^ and MST1 might be colocalized and have competitive effects on FoxO3 transactivation, immunoprecipitation (IP) assays were carried out to define the direct interaction of BRAF^V600E^ and MST1. As shown in Fig. 4A, BRAF^V600E^ was co-precipitated with Flag-tagged MST1 in lysates from 293T cells transfected with BRAF^V600E^ and Flag-tagged MST1 and *vice versa*. To verify the region of MST1 important for the interaction with BRAF^V600E^, IP assays using MST1 deletion constructs, such as MST1 N-terminal (residues 1–326) and C-terminal (327–487) constructs (Fig. 4B), were carried out. These IP assays consistently demonstrated that BRAF^V600E^ binds to the C-terminal region of MST1 (Fig. 4C), which contains of regulatory binding domain with the Sav/RASSF1/Hpo (SARAH) domain of RASSF1A [40], [41], [42]. To verify theBRAF^V600E^mutation specific binding with MST1, we performed additional IP assays using BRAF^WT^ and BRAF^V600E^ constructs. Furthermore, as shown in Fig. 4D, BRAF^V600E^was consistently co-precipitated with Flag-tagged MST1 whereas wild-type BRAF did not. Taken together, these results show that BRAF^V600E^ can bind to the C-terminal domain of MST1 via mutation-specific protein-protein interaction. Because RASSF1A also binds to the C-terminal domain of MST1 and increases MST1 kinase activity, the interaction between BRAF^V600E^ and MST1 might affect MST1 kinase activity by interrupting the heterotypic interaction of MST1 with RASSF1A. To verify this, the effect of BRAF^V600E^ on the kinase activity of MST1 was examined *in vitro* using 293T cells, which were transfected with Flag-MST1 and BRAF^V600E^ as indicated. The immunoprecipitates obtained with an antibody to Flag were used to assay for MST1 kinase activity using histone H2B as a substrate. As seen in Fig. 4E, the expression of BRAF^V600E^ completely abolished MST1 kinase activity. These data suggest that BRAF^V600E^ can negatively regulate MST1 kinase through direct interaction with its C-terminus.

### BRAF^V600E^ generates an anti-apoptotic effect via repression of p21 and p27 induction

**Figure 5:**
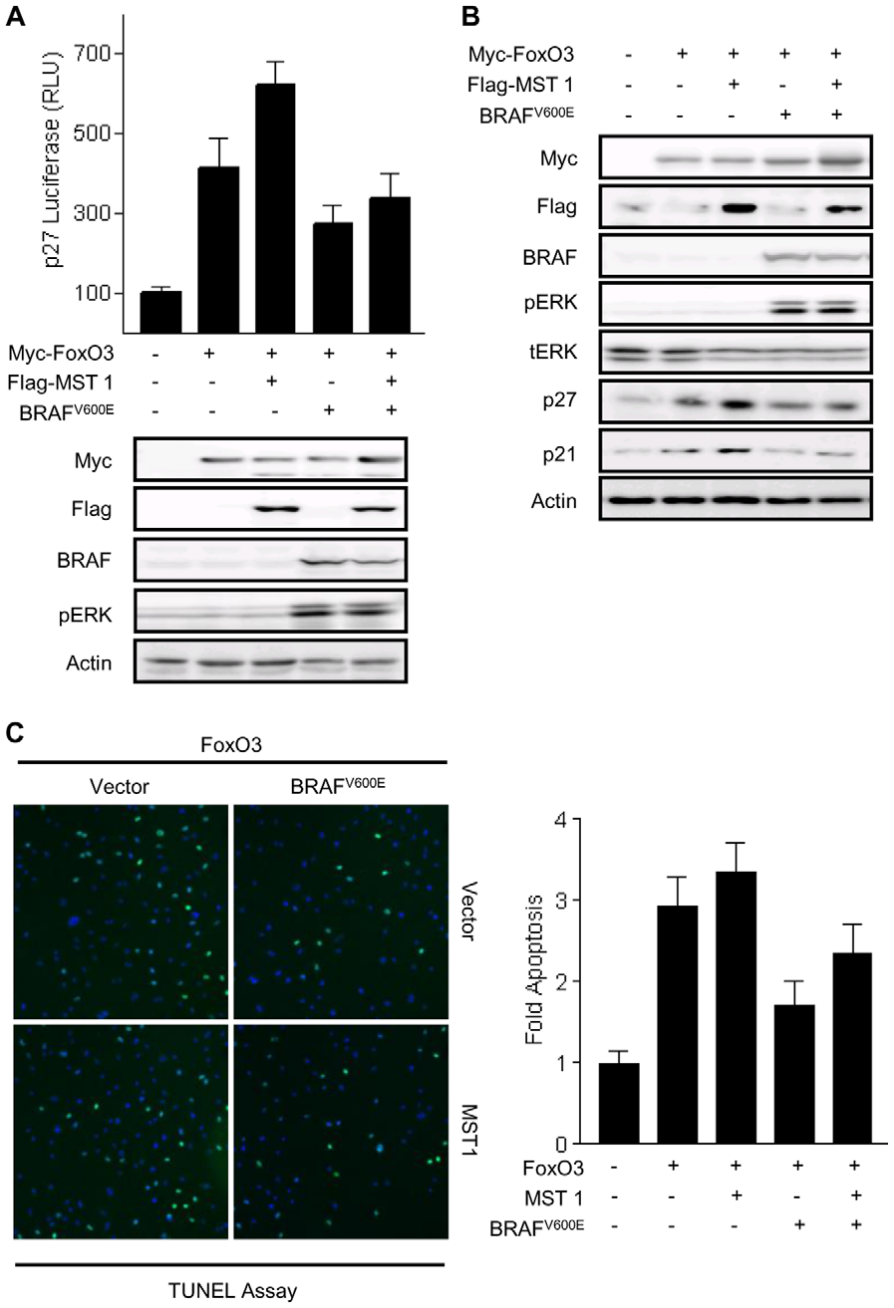
BRAF^V600E^ affects FoxO3 mediated apoptotic gene expression. (a) 293 cells were transiently transfected with *p27*-promoter-luc (0.1 µg/well) and Myc-FoxO3, and co-transfected with plasmids expressing Flag-MST1 as well as plasmids expressing BRAF^V600E^. After 24 h, cells were harvested and analyzed by Western blotting. (b) FRO cells were transiently transfected with Myc-FoxO3 and co-transfected with plasmids expressing Flag-MST1 or BRAF^V600E^ as indicated.Total lysates were immunoblotted with the indicated antibodies. (c) MST1-FoxO-mediated apoptosis is reversed by BRAF^V600E^. 293T cells were transiently transfected with Myc-FoxO3 and co-transfected with Flag-MST1 as well as BRAF^V600E^ as indicated. After 24 hours, FoxO3 and MST-expressing cells were fixed and were then stained by the TUNEL protocol. TUNEL-positive cells (*green*) exhibit condensed or fragmented DAPI-stained nuclei (*blue*), indicative of apoptosis. Quantification of the results is shown in the right panel. MST1 expression significantly increased apoptosis, which is reduced by expression of BRAF^V600E^. For each experiment at least 1000 cells were counted and analyzed by Cellomics'sbioapplication. All data are presented as mean±SD.

FoxO3 acts as a cell-cycle regulator by inducing the transcription of genes such as p21 and p27 [43], the promoters of which contain IRS elements. The results of the assessment of the regulatory effect of BRAF^V600E^ on the p27 reporter are shown in Fig. 5A. FoxO3 increased p27 reporter activity and MST1 potentiated this FoxO3-induced p27 activation. In addition, MST1-FoxO3 mediated p27 reporter activation was abrogated by co-transfection with BRAF^V600E^. To confirm that BRAF^V600E^ can affect p27 reporter activity, the inhibitory effect of BRAF^V600E^ on p21 and p27 induction was investigated by Western blot analysis (Fig. 5B). The results confirmed the MST1-FoxO3 induction of p21 and p27, which was almost completely reversed by BRAF^V600E^ co-transfection. Notably, these two G1-checkpoint CDK inhibitors have been associated with the initiation and progression of human tumors because p21 and p27 bind and inhibit cyclin-CDK complexes, inducing cell cycle arrest and apoptosis. Therefore, the TUNEL assay was used to determine whether MST1-FoxO3 promotes apoptosis and if BRAF^V600E^ reverses the MST1-FoxO3 effect (Fig. 5C). Transient co-expression of FoxO3 and MST1 increased the number of TUNEL positive 293T cells, but this number was decreased by the introduction of BRAF^V600E^ (see also right panel of Fig. 5C for statistical analysis). These results imply that the MST1-FoxO3 pathway induction of G1-checkpoint CDK inhibitors promotes apoptosis, and oncogenic BRAF^V600E^ inhibits the activity of these tumor suppressor systems through the regulation of MST1.

### Loss of MST1 aggravates PTC phenotypes in thyroid-specific transgenic BRAF^V600E^ mice

**Figure 6:**
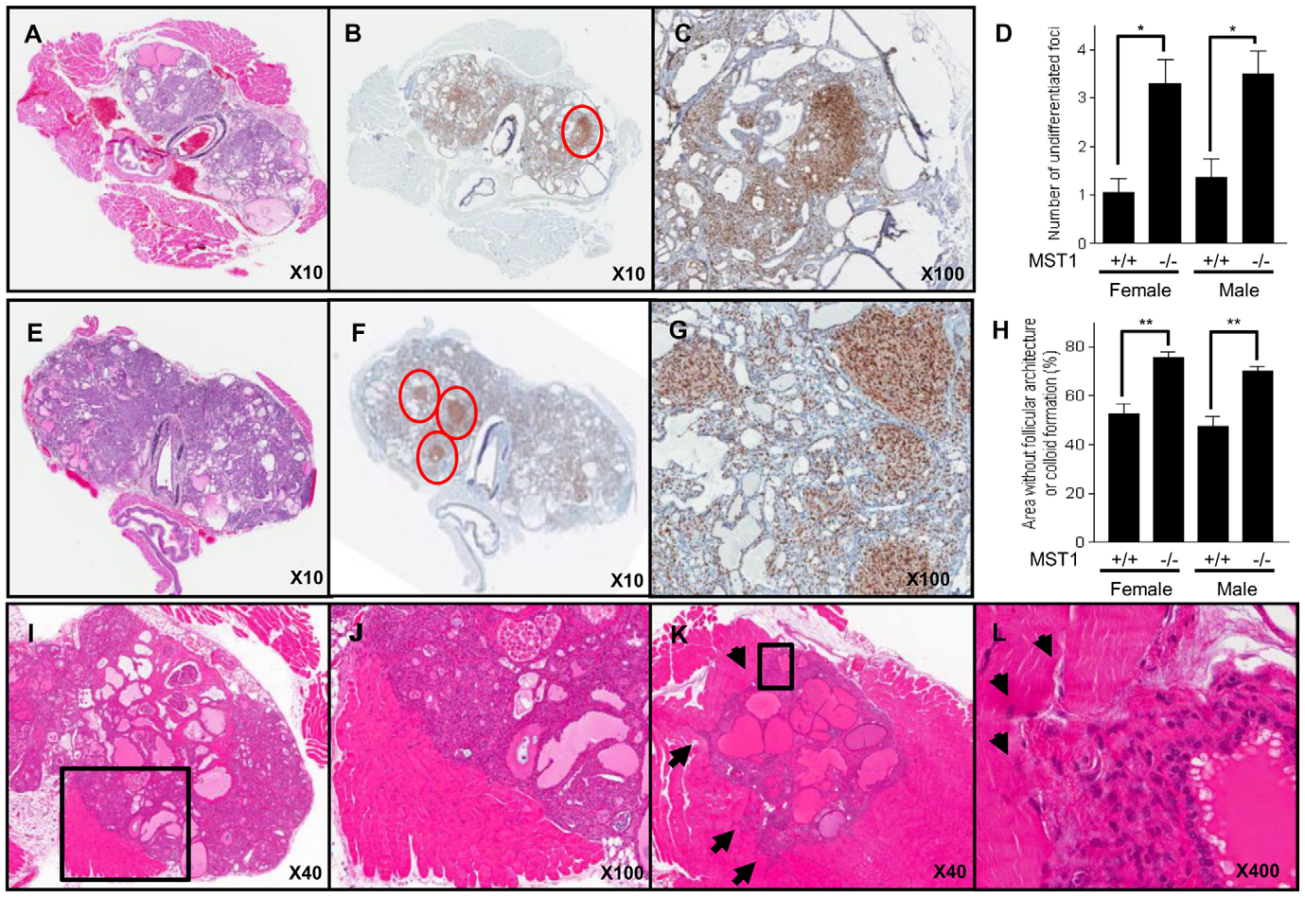
Microscopic features of Tg-BRAF^V600E^/MST1 null mice. Thyroid tissues were fixed in 10% formalin and embedded in paraffin. Five micrometer-thick sections were prepared and stained with H&E. At least five sections from each mouse were used for ImageJ analysis. Differences between Tg-BRAF^V600E^/MST1-null mice and Tg BRAF^V600E^ littermates were assessed by Mann-Whitney U-test. All data are presented as mean±SD. (a) Representative figure from Tg-BRAF^V600E^ littermates shows characteristic features of human PTC, including nuclear enlargement, crowding and overlapping (magnification ×10). (b&c) Representative immunohistochemical staining results with anti-cyclin D1 antibody in Tg-BRAF^V600E^ littermates (b, magnification ×10; c, magnification ×100). Circled area shows intensive staining of cyclin D1 and is magnified in c. (d) Numbers of undifferentiated foci were calculated by counting the number of areas showing increased staining intensity of cyclin D1. (e) Representative image from a Tg-BRAF^V600E^/MST1 null mouse shows a limited area with follicle or colloid formation (e, magnification ×10). (f,g) Representative immunohistochemical staining results with anti-cyclin D1 antibody from a Tg-BRAF^V600E^/MST1 null mouse, suggesting abundant undifferentiated foci (f, magnification ×10; g, magnification ×100). Circled areas show increased staining intensity of cyclin D1 and are magnified in g. (h) ImageJ analysis of estimated areas without follicular architecture or colloid formation. Tg-BRAF^V600E^/MST1 null mice (n = 10) have larger areas than Tg-BRAF^V600E^ littermates (n = 10). (i,j) Representative images from a Tg-BRAF^V600E^/MST1 null mouse show muscle invasion adjacent to the thyroid gland (f, magnification ×40, boxed area is magnified and shown in j (magnification ×100)). (k,l) Representative images from the other Tg-BRAF^V600E^/MST1 null mouse show aggressive invasion to muscle areas surrounding the upper pole of the thyroid gland (k, magnification ×40, boxed area is magnified and presented at l (magnification ×100)). Arrows indicate invasive front in each figure. All data are presented as means±SD: (*) P<0.01; (**) P<0.05 between two groups.

To validate the pathophysiological consequences of the interaction between BRAF^V600E^ and MST1 *in vivo*, animal experiments using thyroid-specific transgenic BRAF^V600E^ mice (Tg-BRAF^V600E^ mice) and MST1 null mice were designed. Tg-BRAF^V600E^ mice were crossed with MST1 null mice and the phenotypic differences between Tg-BRAF^V600E^/MST1 null mice and their Tg-BRAF^V600E^ littermates were analyzed at 16 weeks [4]. The thyroid glands of MST1 null mice with wild-type BRAF were normal (data not shown). However, Tg-BRAF^V600E^/MST1 null mice showed aggressive PTC tumor phenotypes compared with their Tg-BRAF^V600E^ littermates. Although the presence of lymph node metastasis was not observed in either of the two groups, Tg-BRAF^V600E^/MST1 null mice showed abundant foci of undifferentiated carcinomas and large areas without follicular architecture or colloid formation (Figure 6A–H). For the statistical analysis of these results, immunohistochemical staining of cyclin D1 was performed, which showed an intensive cyclin D1 staining area as undifferentiated foci (Fig. 6B&C, F–G). As shown in Figure 6D, the number of undifferentiated foci in female Tg-BRAF^V600E^/MST1 null mice was 3.3±0.86, but that of their Tg-BRAF^V600E^ littermates was 1.05±0.51, reflecting a statistical difference between the two groups (P<0.001). Similarly, male Tg-BRAF^V600E^/MST1 null mice showed a higher number of undifferentiated foci than their Tg-BRAF^V600E^ littermates (3.5±0.83and 1.35±0.67, respectively; P<0.001). The total area composed of sheets of spindle cells without follicular architecture or colloid formation was estimated and ImageJ (http://rsbweb.nih.gov/ij/) was used to quantify these areas and analyze the differences between Tg-BRAF^V600E^/MST1 null mice and their Tg-BRAF^V600E^ littermates (Figure 6H). In female mice, Tg-BRAF^V600E^/MST1 null mice had a significantly larger area without follicular architecture or colloid formation than their Tg-BRAF^V600E^ littermates (75.47±3.83% and 52.69±7.93%, respectively; P = 0.034). In male mice, Tg-BRAF^V600E^/MST1 null mice also showed a larger area without follicular architecture or colloid formation compared to their Tg-BRAF^V600E^ littermates (70.02±3.68% and47.31±7.93%, respectively; P = 0.021). This *in vivo* study suggests that the direct interaction between BRAF^V600E^ and MST1 could affect the behavior of papillary thyroid cancer. Another piece of evidence demonstrating the aggressive tumor phenotype in these Tg-BRAF^V600E^/MST1 null mice was the frequent presence of muscle invasion (Figure 6I–L), which was not observed in their Tg-BRAF^V600E^ littermates.

## Discussion

BRAF^V600E^ constitutively activates the MEK-ERK signaling pathway, promoting ERK-dependent transcriptional activity, which regulates cell growth, cell proliferation, apoptosis evasion, replicative potential, angiogenesis, and tissue invasion [44], [45]. RASSF1A primarily interacts with small GTPases such as Ras to facilitate cell cycle arrest and apoptosis. Transcriptional silencing of RASSF1A by promoter methylation has been reported in many human cancers including thyroid cancer [46], [47]. However, epigenetic alteration has been regarded as a slow and time-consuming process for gene silencing, implying that tumor suppressors such as RASSF1A are inhibited to allow the initiation and maintenance of oncogenic processes necessary for the establishment of clinically significant thyroid cancers [48]. The results of the present study suggest that BRAF^V600E^ tumors might regulate RASSF1A function via a novel mechanism. RASSF1A transfection induced p21 and p27 in a MST1-dependent manner, suggesting that the RASSF1A-MST1-FoxO3 pathway can induce cell cycle arrest. In addition, BRAF^V600E^ suppressed the RASSF1A-MST1-mediated signal, inhibiting p21 and p27 induction, supporting the hypothesis of the present work. It has been suggested that BRAF^V600E^-activated ERK inhibits FoxO3 by murine double minute 2 (MDM2)-mediated degradation [25]. However, BRAF^V600E^ did not alter HA-FoxO3 protein levels, as shown by Western blot analysis, and, interestingly, treatment with a MEK inhibitor did not abolish the BRAF^V600E^-mediated inactivation of FoxO3. In the present study, BRAF^V600E^ did not induce phosphorylation of Akt/PKB and, furthermore, the addition of PI3 kinase inhibitors did not affect the inhibition of FoxO3 by BRAF^V600E^. Therefore, BRAF^V600E^ did not affect FoxO3 transactivation by a PI3-Akt/PKB-dependent signaling pathway, whereas RET/PTC inactivates FoxO3 by Akt/PKB activation [23]. In addition, the silencing of RAF-1, which has been reported to bind with MST2, did not alter the inhibition of FoxO3 transactivation by BRAF^V600E^.Taken together,these data suggest that FoxO3 inactivation might be a common process in the carcinogenesis of PTC and that BRAF^V600E^ inhibits FoxO3 transactivation through a MEK-ERK–, Akt/PKB–, or RAF-1–independent pathway. The results of the present study suggest the existence of a novel FoxO3 inhibitory mechanism that might operate in BRAF^V600E^-induced PTC.

MST1 kinase had a counter-regulatory effect on FoxO3 activation and localization compared with Akt/PKB [49], [50]. In contrast to Akt/PKB, MST1 phosphorylation of FoxO3 promotes its nuclear translocation, thereby inducing cell death [19]. A novel inhibitory mechanism of FoxO3 transactivation might be operational in BRAF^V600E^-induced PTC, which led to the evaluation of BRAF^V600E^ regulation of MST1-FoxO3 signaling pathways. A reporter assay revealed that MST1-induced FoxO3 transactivation was completely inhibited by co-transfection with BRAF^V600E^. In addition, over-expression of MST1 prevented BRAF^V600E^-induced FoxO3 inactivation. These data suggested that cross-regulation between MST1 and BRAF^V600E^ might play a critical role in regulating FoxO3 transactivation. Supporting this idea, immunofluorescence studies revealed not only the BRAF^V600E^-mediated inhibition of MST1-induced FoxO3 nuclear translocation, but also a direct interaction between MST1 and BRAF^V600E^, which was confirmed through IP assays that demonstrated that BRAF^V600E^ binds to the C-terminal dimerization domain of MST1.

In fact, the dimerization domain of MST1/2 mediates MST1 kinase activity by interacting with binding partners such as RASSF1A and hWW45 [20], [51]. Because RASSF1A promotes MST1 activation and apoptosis via this hetero-dimerization, we postulated that the direct interaction of MST1 with BRAF^V600E^ might affect MST1 kinase activity. Actually, our *in vitro* kinase assay clearly showed that MST1 kinase activity on histone H2B was markedly decreased by BRAF^V600E^. Another possible mechanism of MST1 regulation may be the inhibitory phosphorylation of MST1 by BRAF^V600E^. Prior research has shown that Akt/PKB can phosphorylate the Thr^387^ residue of MST1 and prevent its proteolytic activity, providing a mechanism for the Akt/PKB-mediated inactivation of MST1-FoxO3 pathways [50]. Although the BRAF^V600E^-induced MST1 phosphorylation was not assessed, it remains possible that BRAF^V600E^ may phosphorylate and inactivate MST1 through direct interaction with the C-terminal domain of MST1.

Few mouse models exist for the study of tumor suppressor systems in PTC. In the present study, a new *in vivo* model was generated to investigate the cross-regulation between BRAF^V600E^ and MST1, and the results obtained with transgenic mice were consistent with the *in vitro* generated data. Although MST1 knockout mice did not show any thyroid gland pathological changes compared to wild-type mice, Tg-BRAF^V600E^/MST1 null mice showed an aggressive phenotype, supporting the hypothesis that MST1 might have a modifying effect on BRAF^V600E^-induced carcinogenesis. Although the lack of an appropriate antibody for the specific immunohistochemical detection of serine 207 residue in FOXO3 phosphorylated by MST1 kinaseprevented the detection of FoxO3 activity modified by MST1, these results, coupled with the results of *in vitro* assays, suggest that BRAF^V600E^ may alleviate the tumor suppressor function of RASSF1A-MST1 and *vice versa*.

In conclusion, the present study suggests that a direct interaction between oncogenic BRAF^V600E^ and MST1 kinase plays a crucial role in determining tumor behavior in PTC. This novel interaction might provide advanced insight into the pathogenesis of the disease, and form the basis for novel drug development for the treatment of BRAF^V600E^-induced PTC. In light of this, it will be important to delineate the precise regulatory mechanism of the RASSF1A-MST1 pathway and verify the action of BRAF^V600E^ on this signal pathway in human PTC.

## Supporting Information

Figure S1 MST1 significantly increases 3XIRS reporter activities. (A) 293T cells were cultured in 12 well dishes until they reached 80% confluence, and co-transfected with 3XIRS Luc (100 ng/well), FoxO3 (0.5 µg/well), and MST1 (0.5 µg/well) as indicated for 24 hours. To verify transfection efficacy, total lysates were immunoblotted with anti-HA, anti-Flag, and anti-Actin antibodies. (B) Cells were co-transfected with 3XIRS Luc (100 ng/well), FoxO3 (0.5 µg/well), and MST1 (indicated amount/well). Total lysates were immunoblotted with anti-HA, anti-Flag, and anti-Actin antibodies. For each sample, firefly luciferase activity was normalized to *Renilla* luciferase activity and expressed as relative-fold change compared to basal luciferase activity. All data are presented as mean±SD: (*) P<0.01 between two groups.
(TIF)

Figure S2 BRAF^V600E^ inhibits MST1-induced nuclear translocation of FoxO1. 293T cells were cultured in a six-well dish until they reached 80% confluence and co-transfected with FoxO3-GFP (0.5 µg/well), Flag-MST1 (0.5 µg/well), and Myc-BRAF^V600E^ (0.5 µg/well) as indicated. Twenty-four hours after transfection, the cells were prepared for subcellular fractionation using the Nuclear/Cytosol Fractionation kit (BioVision, Inc. CA). The markers, origin recognition complex subunit 1 (ORC1) and β-tubulin, were used to verify the identity and purity of the nuclear and cytosolic fractions, respectively. Based on these markers, a good overall yield was obtained without mixing of the fractions.
(TIF)

Figure S3 BRAF^V600E^ mediated FoxO3 inhibition was not altered by RAF-1. 293T cells were cultured in 12 well dishes until they reached 80% confluence and co-transfected with 3XIRS Luc (100 ng/well), FoxO3 (0.5 µg/well), BRAF^V600E^ (0.5 µg/well), and SiRAF-1 (20 µM/well Stealth™ RNA) for 24 h as indicated. Total lysates were immunoblotted with anti-HA, anti-BRAF, anti-RAF-1, and anti-Actin antibodies. For each sample, firefly luciferase activity was normalized to *Renilla* luciferase activity and expressed as relative-fold change compared to basal luciferase activity. All data are presented as mean±SD: (*) P<0.01 between two groups.
(TIF)

Figure S4 BRAF^V600E^ suppresses FoxO3 transactivation via a MEK/ERK-, PI3 kinase-independent pathway. 293T cells were cultured in 12 well dishes until they reached 80% confluence, and co-transfected with 3XIRS Luc (100 ng/well), FoxO3 (0.5 µg/well), and BRAF^V600E^ (0.5 µg/well) as indicated for 24 h. MEK inhibitor (lane 3, U0126 20 µM/well) and PI3 kinase inhibitors (lane 4, Wortmannin 200 nM/well, and lane 5, LY294002 20 µM/well) were added. Total lysates were immunoblotted with anti-HA, anti-BRAF, anti-pERK, anti-ERK, anti-pAkt/PKB, anti-Akt/PKB, and anti-Actin antibodies. For each sample, firefly luciferase activity was normalized to *Renilla* luciferase activity and expressed as relative fold change compared to basal luciferase activity. All data are presented as mean±SD. Abbreviations: U0, U0126; WT, Wortmannin; LY, LY294002; and Con, control.
(TIF)
