# Supplementary figures and images for: Cross-Regulation between Oncogenic BRAFV600E Kinase and the MST1 Pathway in Papillary Thyroid Carcinoma
Source: PLoS One. 2011 Jan 13;6(1):e16180. doi: 10.1371/journal.pone.0016180 (PMC3020965; doi:10.1371/journal.pone.0016180)

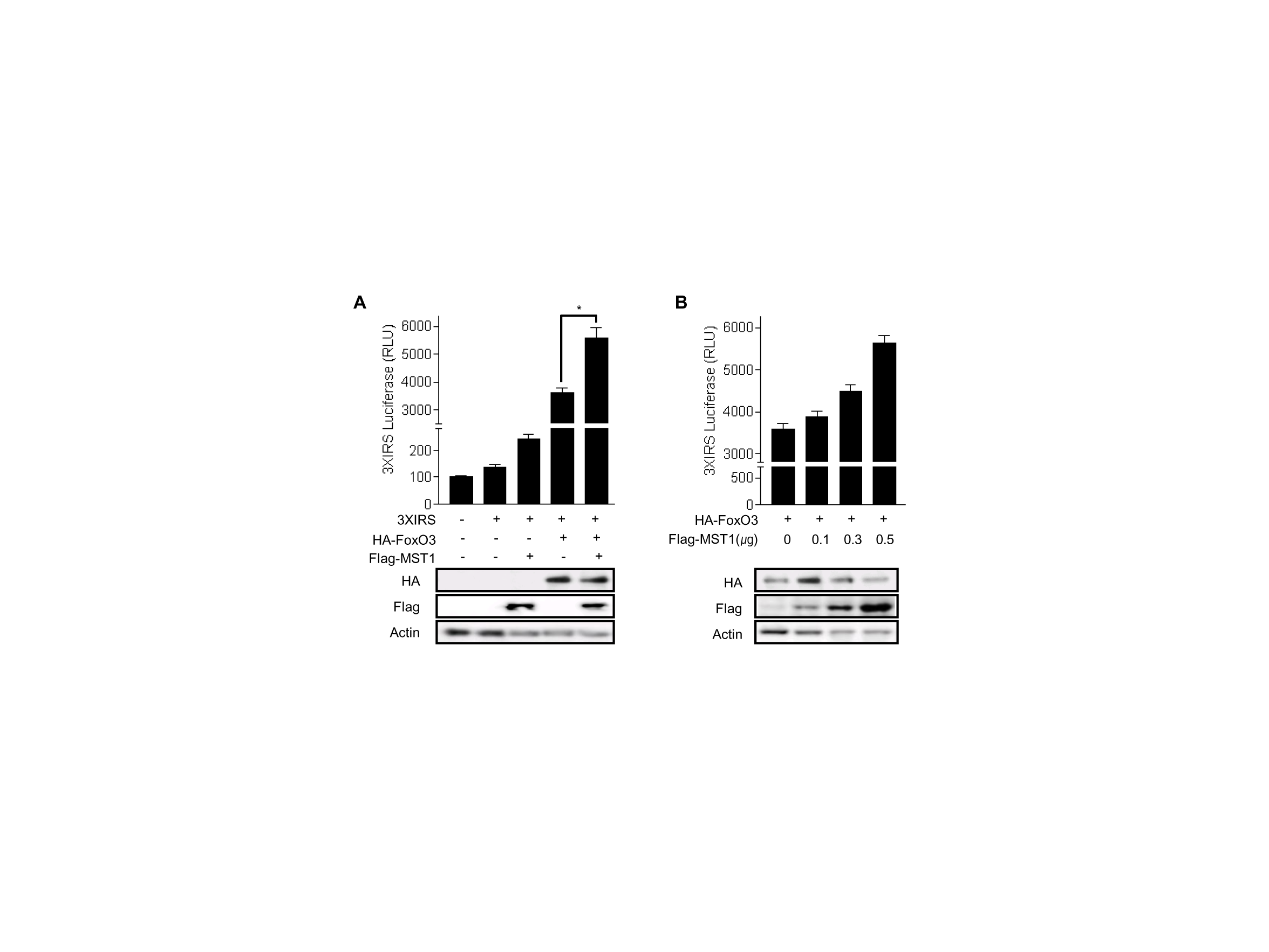

Supplement: Figure S1 — MST1 significantly increases 3XIRS reporter activities. (A) 293T cells were cultured in 12 well dishes until they reached 80% confluence, and co-transfected with 3XIRS Luc (100 ng/well), FoxO3 (0.5 µg/well), and MST1 (0.5 µg/well) as indicated for 24 hours. To verify transfection efficacy, total lysates were immunoblotted with anti-HA, anti-Flag, and anti-Actin antibodies. (B) Cells were co-transfected with 3XIRS Luc (100 ng/well), FoxO3 (0.5 µg/well), and MST1 (indicated amount/well). Total lysates were immunoblotted with anti-HA, anti-Flag, and anti-Actin antibodies. For each sample, firefly luciferase activity was normalized to Renilla luciferase activity and expressed as relative-fold change compared to basal luciferase activity. All data are presented as mean±SD: (*) P<0.01 between two groups. (TIF) [file pone.0016180.s001.tif]

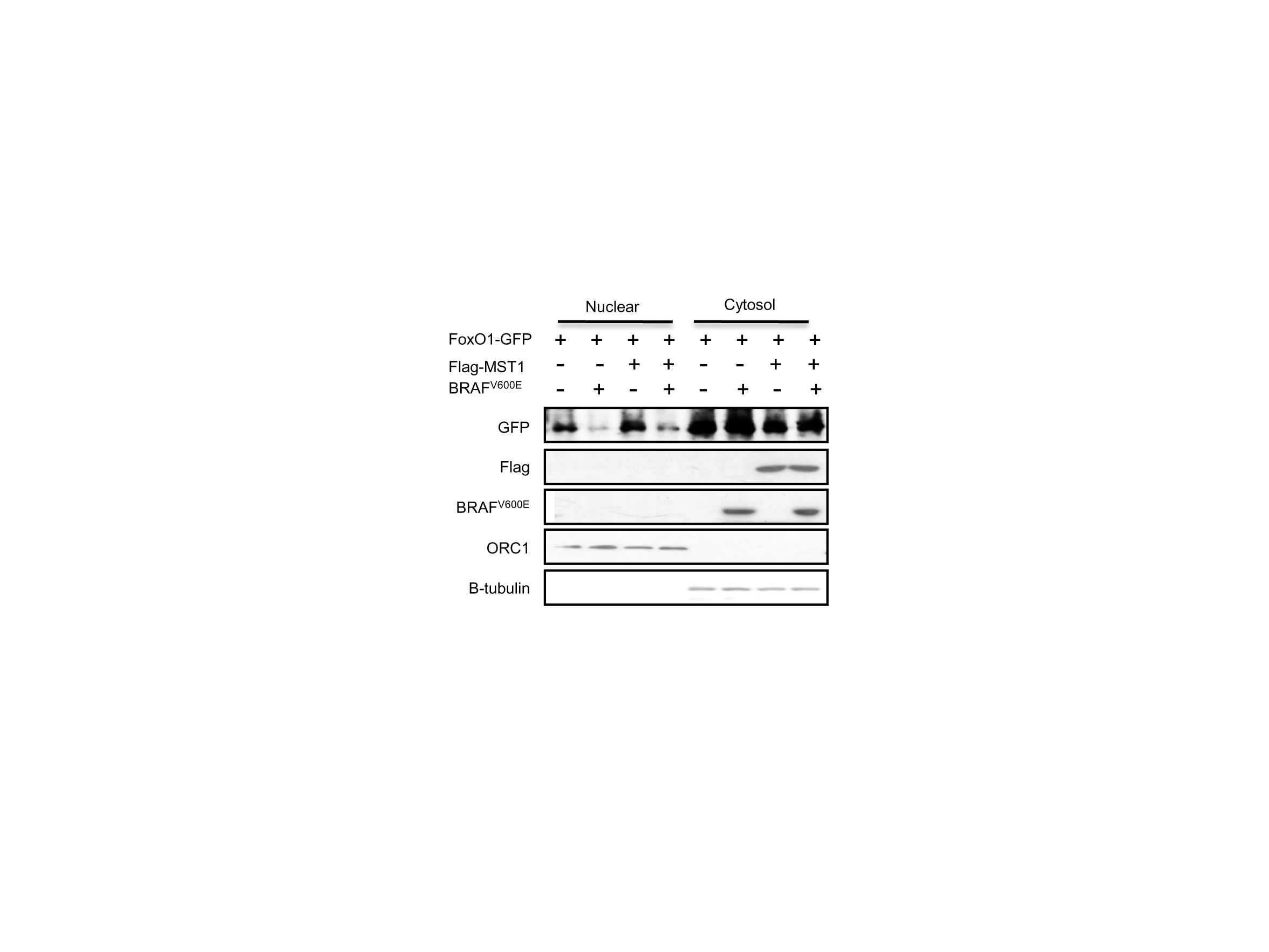

Supplement: Figure S2 — BRAFV600E inhibits MST1-induced nuclear translocation of FoxO1. 293T cells were cultured in a six-well dish until they reached 80% confluence and co-transfected with FoxO3-GFP (0.5 µg/well), Flag-MST1 (0.5 µg/well), and Myc-BRAFV600E (0.5 µg/well) as indicated. Twenty-four hours after transfection, the cells were prepared for subcellular fractionation using the Nuclear/Cytosol Fractionation kit (BioVision, Inc. CA). The markers, origin recognition complex subunit 1 (ORC1) and β-tubulin, were used to verify the identity and purity of the nuclear and cytosolic fractions, respectively. Based on these markers, a good overall yield was obtained without mixing of the fractions. (TIF) [file pone.0016180.s002.tif]

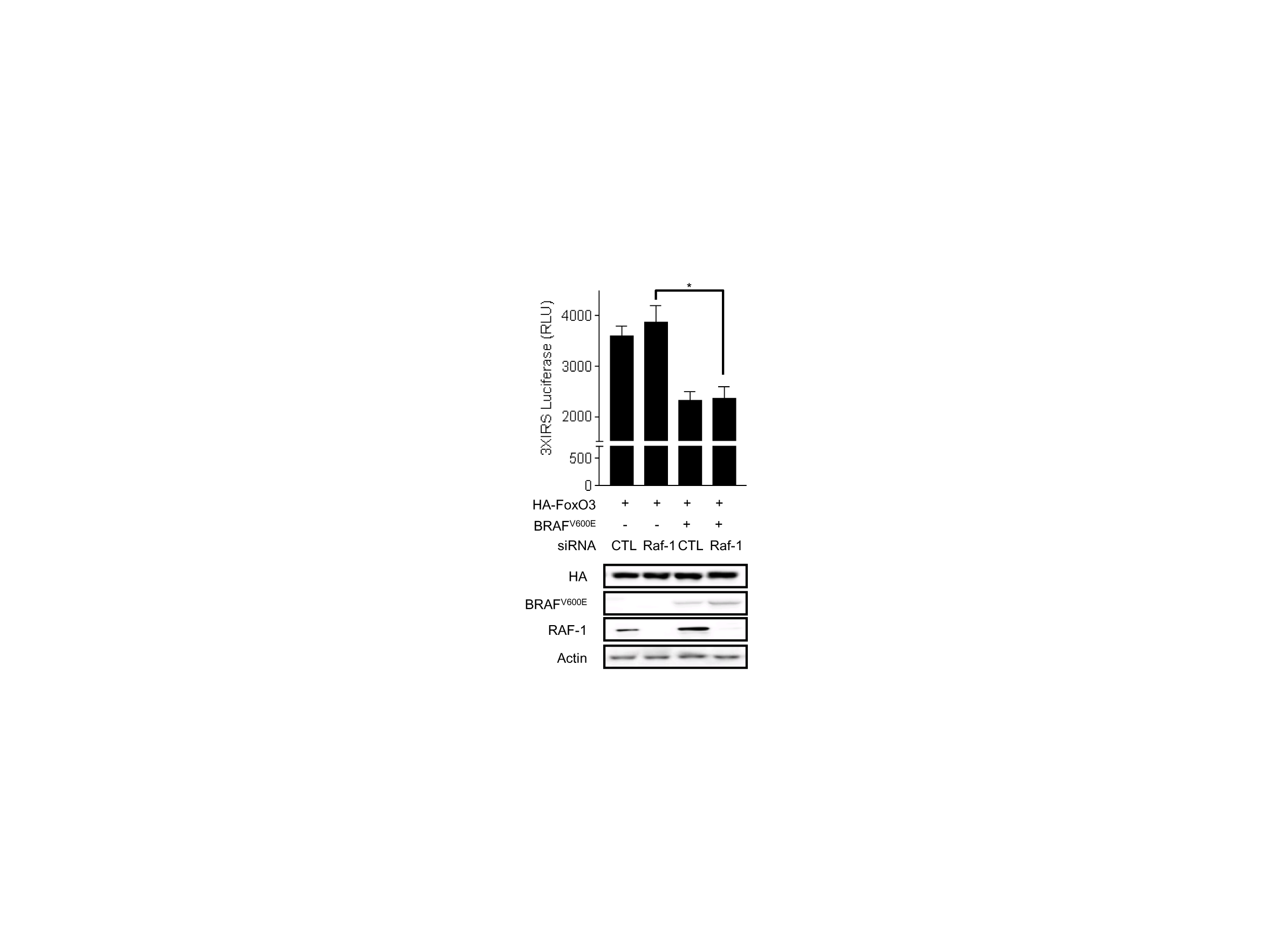

Supplement: Figure S3 — BRAFV600E mediated FoxO3 inhibition was not altered by RAF-1. 293T cells were cultured in 12 well dishes until they reached 80% confluence and co-transfected with 3XIRS Luc (100 ng/well), FoxO3 (0.5 µg/well), BRAFV600E (0.5 µg/well), and SiRAF-1 (20 µM/well Stealth™ RNA) for 24 h as indicated. Total lysates were immunoblotted with anti-HA, anti-BRAF, anti-RAF-1, and anti-Actin antibodies. For each sample, firefly luciferase activity was normalized to Renilla luciferase activity and expressed as relative-fold change compared to basal luciferase activity. All data are presented as mean±SD: (*) P<0.01 between two groups. (TIF) [file pone.0016180.s003.tif]

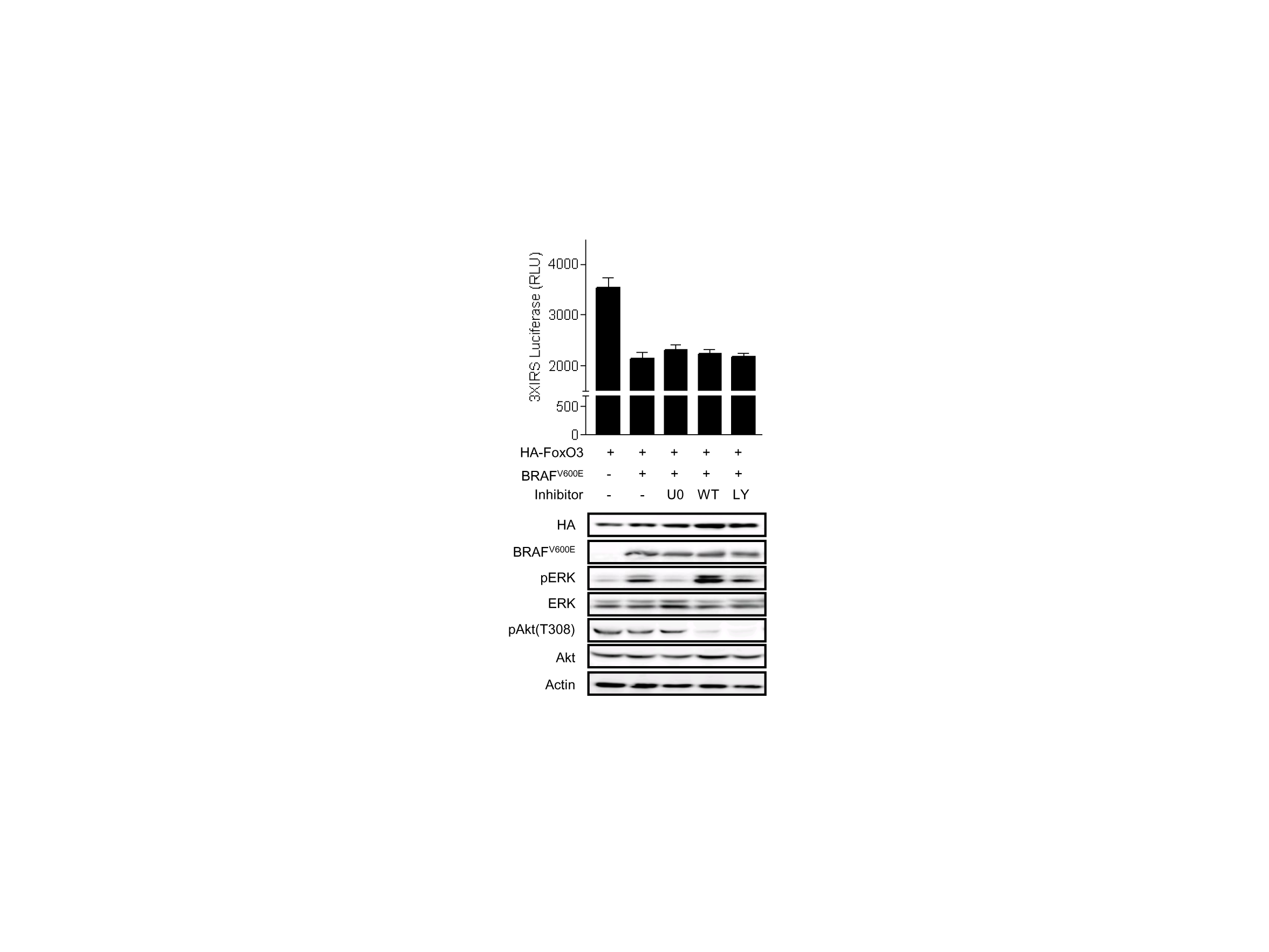

Supplement: Figure S4 — BRAFV600E suppresses FoxO3 transactivation via a MEK/ERK-, PI3 kinase-independent pathway. 293T cells were cultured in 12 well dishes until they reached 80% confluence, and co-transfected with 3XIRS Luc (100 ng/well), FoxO3 (0.5 µg/well), and BRAFV600E (0.5 µg/well) as indicated for 24 h. MEK inhibitor (lane 3, U0126 20 µM/well) and PI3 kinase inhibitors (lane 4, Wortmannin 200 nM/well, and lane 5, LY294002 20 µM/well) were added. Total lysates were immunoblotted with anti-HA, anti-BRAF, anti-pERK, anti-ERK, anti-pAkt/PKB, anti-Akt/PKB, and anti-Actin antibodies. For each sample, firefly luciferase activity was normalized to Renilla luciferase activity and expressed as relative fold change compared to basal luciferase activity. All data are presented as mean±SD. Abbreviations: U0, U0126; WT, Wortmannin; LY, LY294002; and Con, control. (TIF) [file pone.0016180.s004.tif]
